# PyDapsys: an open-source library for accessing electrophysiology data recorded with DAPSYS

**DOI:** 10.3389/fninf.2023.1250260

**Published:** 2023-09-14

**Authors:** Peter Konradi, Alina Troglio, Ariadna Pérez Garriga, Aarón Pérez Martín, Rainer Röhrig, Barbara Namer, Ekaterina Kutafina

**Affiliations:** ^1^Institute of Medical Informatics, Medical Faculty, RWTH Aachen University, Aachen, Germany; ^2^Research Group Neuroscience, IZKF, RWTH Aachen, Aachen, Germany; ^3^Simulation and Data Lab Neuroscience, Jülich Supercomputing Centre (JSC), Institute for Advanced Simulation, JARA, Forschungszentrum Jülich GmbH, Jülich, Germany; ^4^Department for Neurophysiology, University Hospital RWTH Aachen, Aachen, Germany; ^5^Institute of Physiology and Pathophysiology, Friedrich-Alexander-Universität Erlangen-Nürnberg, Erlangen, Germany

**Keywords:** interoperability, open data, FAIR, data management tools, reverse-engineered, microneurography, electrophysiology, pain

## Abstract

In the field of neuroscience, a considerable number of commercial data acquisition and processing solutions rely on proprietary formats for data storage. This often leads to data being locked up in formats that are only accessible by using the original software, which may lead to interoperability problems. In fact, even the loss of data access is possible if the software becomes unsupported, changed, or otherwise unavailable. To ensure FAIR data management, strategies should be established to enable long-term, independent, and unified access to data in proprietary formats. In this work, we demonstrate PyDapsys, a solution to gain open access to data that was acquired using the proprietary recording system DAPSYS. PyDapsys enables us to open the recorded files directly in Python and saves them as NIX files, commonly used for open research in the electrophysiology domain. Thus, PyDapsys secures efficient and open access to existing and prospective data. The manuscript demonstrates the complete process of reverse engineering a proprietary electrophysiological format on the example of microneurography data collected for studies on pain and itch signaling in peripheral neural fibers.

## 1. Introduction

Many commercial software solutions use custom proprietary formats to store their data. Reasons vary from dealing with special use cases to trying to lock users into a vendor-specific ecosystem. While there is a trend in the general IT space to open-source custom solutions and establish cross-vendor standards (Kilamo et al., 2012), in the scientific world, the focus is put on FAIR data principles (Wilkinson et al., 2016). FAIR principles consist of a number of requirements for data to be findable, accessible, interoperable, and reusable. Proprietary formats naturally obstruct the adoption of these principles. In some research domains, large efforts are put into building solutions to convert proprietary formats into open standards, while simultaneously lobbying companies to use open formats. Examples of such formats are DICOM^1^ for storing, managing, and exchanging medical images and EDF (Kemp et al., 1992) for biosignals, including EEG systems.

Progress has also been made in the field of neuroscience, where the Neuroscience Information Exchange format (NIX) (Stoewer et al., 2014) and the Neurodata Without Borders (NWB) (Rübel et al., 2022) projects are aiming to establish community standards for sharing neuroscientific data. Both projects specify a storage layout, which is implemented on top of the Hierarchical Data Format (HDF5) but use different approaches to model data. NWB uses a stricter and more standardized data model, whereas NIX allows for a comparatively flexible structure and can describe the file contents using the open metadata Markup Language (odML) (Grewe et al., 2011).

However, smaller fields of neuroscience are facing challenges to fully adopt FAIR data principles, as vendors may not have the resources to address the specific wishes of such small user-bases. The “Data Acquisition Processor System” (DAPSYS)^2^ is a general-purpose neurophysiological data acquisition system (DAS) for recording and processing neural signals, which is, among other places, used in the microneurography (MNG) lab of the University Hospital RWTH Aachen. MNG is an electrophysiological technique to record activity from single nerve fibers of the peripheral nervous system using a single microelectrode (Vallbo and Hagbarth, 1968; Torebjork and Hallin, 1974; Ackerley and Watkins, 2018). Due to the small size of the electrode, the method causes only minimal discomfort and does not require anesthetics. This means that the volunteer stays awake and cooperates during the recording, making it possible to correlate nerve fiber signals with individual sensations. Thus, MNG is a unique translational method in sensory research in humans, especially in chronic pain and itch.

DAPSYS uses a proprietary format to store data and only offers manual (file-by-file) export of the recordings to CSV files. However, the CSV exports produce comparatively large files (see Table 1) and take a long time (see Table 2). In addition, some minor precision loss due to the fixed number of decimals in the exported CSV is observed. Our recent works on establishing data-sharing standards in the MNG community and developing a computational pipeline for spike analysis in MNG data (Schlebusch et al., 2021; Kutafina et al., 2022; Troglio et al., 2023) has raised the urgency for an efficient way to read DAPSYS recordings and store them in more suitable data formats, such as HDF5.

**Table 1:** The size difference between CSV files created by the DAPSYS export and the files created by using PyDapsys with the NIX-exporter of the Neo library.

Original file size [MiB]	CSV file size [MiB]	NIX/H5 file size [MiB]	Size increase CSV [%]	Size increase NIX/H5 [%]
44.5	229.5	45.1	415.73	1.35
109.0	576.2	109.7	428.62	0.64
124.9	664.5	126.1	432.83	0.96
165.8	889.2	167.4	436.31	0.97

**Table 2:** The time comparison for exporting the continuous recording.

Original file size [MiB]	CSV export time* [s]	PyDapsys export to NIX/H5 time* [s]	Speedup PyDapsys vs. CSV export*	PyDapsys total time [s]
44.5	35	0.36	97.2	0.77
109.0	91	0.46	197.8	0.84
124.9	102	0.68	150.0	1.03
165.8	133	0.98	135.7	1.49

*Time required for processing and writing, excluding user interaction.

While there are many commercial applications for reverse engineering, most of them target computer science professionals and the primary use-case of reverse engineering software, not file formats. The MARBLE project^3^ is to our best knowledge the first research-oriented solution to reverse engineer file formats with the aim of making the process as accessible as possible. However, at the time of the reported work, MARBLE was still in development and the usage required problem-specific adjustments.

Therefore, in this paper, we show our approach to reverse engineering the DAPSYS file format and implement a Python library to gain open access to our own data recorded in the microneurography lab. By providing functionality to load data into the structure defined by the Neo library (Garcia et al., 2014), it can be simply exported to multiple data formats used in electrophysiology, including NIX. This ensures full access to the data even if DAPSYS is unavailable.

The primary aim of our work is to ensure the accessibility and interoperability of DAPSYS-recorded data sets. The secondary aim is to share the steps of our reverse engineering solution with the neuroscience community to support building FAIR access to rare data formats.

## 2. Method

### 2.1. Data

We used four DAPSYS files, recorded at the microneurography labs of the University Hospital RWTH Aachen and Friedrich-Alexander-University of Erlangen-Nürnberg. The studies involving human participants were reviewed and approved by the Ethics Boards of those two institutions with the corresponding numbers EK141-19 and 4361. The participants provided their written informed consent, and the studies were conducted according to the Declaration of Helsinki.

### 2.2. Reverse engineering method

**Figure 1:**
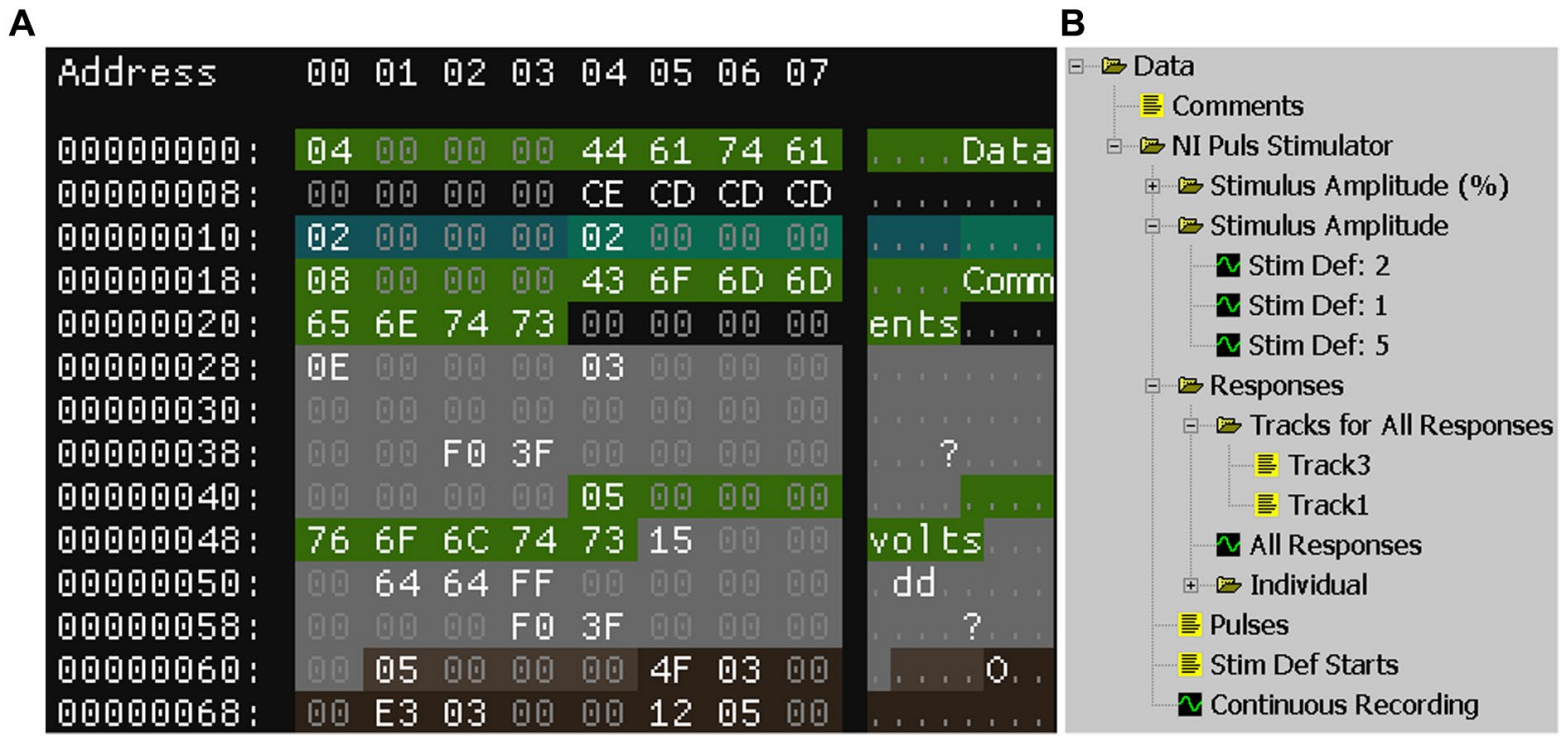
**(A)** “ImHex” showing the start of a DAPSYS files’ table of contents section. Shown are the hexadecimal byte-values of the respective address and the interpretation of that byte as characters. Data fields of structures are shown in different colors. The address shown is in relation to the start of the table of contents. **(B)** Structure of the same file from panel **(A)** shown by the GUI of DAPSYS.

For the reverse engineering process, we used the hex editor “ImHex”^4^ to open and analyze the DAPSYS files. A hex editor shows the binary contents of a file in hexadecimal representation. A value of a single byte can be represented by only two characters, making it easier to recognize patterns (see Figure 1A for an example). Since we knew what values the file should contain, we were able to search for them and identify related fields. From there on, we identified structures based on repeating patterns.

The functions of data fields in the structures were then identified by using the following workflow:

1. Make changes to the file using the DAPSYS GUI (for example: changing the plot configuration, removing data points, etc.).
2. Track these changes DAPSYS made to the binary file and identify changed fields in the hex editor.
3. Open a different recording in the hex editor, identify the known fields, and change their values using the hex editor.
4. Open the changed file from step 3 in DAPSYS and verify that the changes made to the recording fit with the assumed function of the field.

This process was substantially supported by built-in “ImHex” functions like the pattern language that can be used to specify the layout of structures in the binary file. These structures can be utilized to highlight and verify known structures and fields in the file.

### 2.3. Concept of the library implementation

Based on the results from the reverse-engineering process, we implemented a Python library capable of opening and processing recordings. The library also offers a method to export data from DAPSYS recordings into HDF5 files using the NIX structure (abbreviated as NIX/H5) for easier data exchange between labs and software.

#### 2.3.1. Verification

To verify the implementation of the file format in PyDapsys, we read each of the four DAPSYS files (see 2.1) with PyDapsys. The read values were then compared to the CSV files. As the values in the exported CSV files only have limited precision (6 or 4 decimal places, depending on the type of data exported), we first rounded the values read from the file to the same precision before comparing them. Comparison of floating-point values was done by comparing the absolute difference of two values to the system epsilon for 64-bit floating point (f64) values. Numeric values from the CSV were converted to f64 values using built-in Python functions. When comparing f64 with 32-bit floating point (f32) values, the f32 values were first converted to f64. Texts were compared with built-in Python functions.

#### 2.3.2. Performance testing

We also compared the performance (duration and file sizes) of the CSV export of DAPSYS and the export to NIX/H5 using PyDapsys. To achieve comparable measurements, we only looked at the time each system required to write the continuous recording to their respective target format, without the time required for user interactions or loading the data. We had to focus on a single data stream, as DAPSYS would require user interactions in between exporting multiple streams. We chose to focus on the continuous recording, as it makes up the largest part of a file’s size. We also excluded loading times, as there was no reliable way to measure them for DAPSYS. Times for PyDapsys were measured using the wall-clock time directly in the Python program, whereas DAPSYS times were taken by a stopwatch. All measurements were performed on the same system.

## 3. Results

### 3.1. Analysis of the DAPSYS file structure

The DAPSYS user interface displays the contents of a file in a hierarchical structure, composed of folders, text streams, and data streams (see Figure 1B). DAPSYS binary files store data in a flat structure that can be split into 4 parts:

1. Header. Files begin with a header with a fixed length. Information in the header is not required to read the file contents.
2. Data Pages. DAPSYS stores data in discontinuous chunks, which we call “pages.” All pages have a unique ID in the context of the file and can hold either data of a waveform or textual data.
3. Table of Contents (ToC). After the last data page, the ToC begins. It defines the hierarchical structure shown in the GUI and comprises of folders, which can have additional child elements and streams. Streams contain an array of data page IDs.
4. Footer. After the ToC, there comes a small footer consisting of a string holding the version and the serial number of the DAPSYS program used to create the file.

#### 3.1.1. Data pages

**Figure 2:**
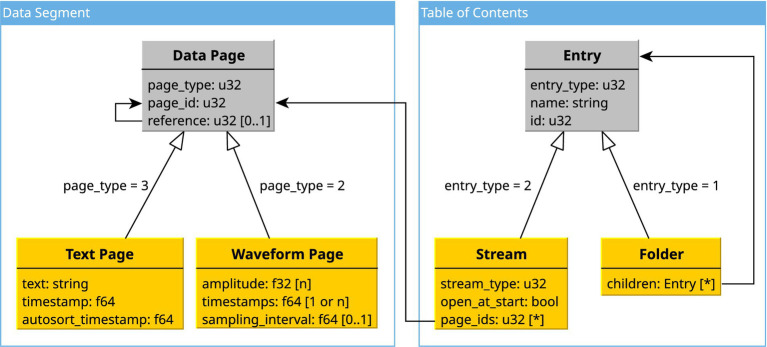
Simplified logical model of a DAPSYS file. Black arrows show to which other fields the value refers. Large unfilled arrows indicate that an entry extends another one. Gray types define shared header fields for a group of types, with extending arrows indicating the field and the value to identify the group of fields that follow the header (e.g., a “Folder” and a “Stream” both start with the fields of an generic “Entry.” Depending on the value of the field “entry_type,” the fields following the “Entry” will be either those of a “Stream” or a “Folder”).

As seen in Figure 2, DAPSYS uses two types of pages: one for waveform data and one for textual data. Both types start with the same fields that store metadata, such as their ID, which is unique among all pages in a file, an identifier for their type (text or waveform), and an optional reference to another page. Waveform pages store the amplitude of the waveform as an array of 32-bit floating point (f32) values, and corresponding timestamps as an array of 64-bit floating point (f64) values. For regularly sampled waveforms, only the first timestamp is saved in the array, while an additional f64 value is used for the regular sampling interval. Text pages are used to store comments as well as sorted spikes. They consist of a string containing the text, and two f64 values. The first f64 value is used to store the timestamp. The second one is used for sorted spikes to indicate the timestamp of the automatically recognized spike. For normal comments, it is set to the same value as the first timestamp. From our observations, DAPSYS writes pages in the order they occur during the recording. If, for example, a comment is entered during a recording, DAPSYS will save the recorded data up to that point in a waveform page, append it to the list of pages followed by the text page containing the comment, and then begin a new waveform page with the new data.

#### 3.1.2. Table of contents

The ToC defines the logical structure of a DAPSYS file. As seen in Figure 2, its elements consist of folders and streams, all of which have an ID unrelated to the IDs used for pages and a string containing their display name. Folders can have several other elements as children. Streams contain multiple fields for storing the configuration of the plot used to visualize their data and most importantly, contain an array of the page IDs belonging to that stream. A stream may either reference text pages or waveform pages, making it a text or data stream, respectively.

### 3.2. Development of the Python library “PyDapsys”

The functionality of PyDapsys [see (Konradi et al., 2023) for the repository containing the source code. The package is also available on PyPI as “pydapsys”] focuses on accessing data stored in a DAPSYS file. Pages are read into a dictionary that maps the page IDs to an object storing the metadata (type of the page, ID, optional ID of the referenced page) and data, i.e., text and timestamps for text pages of the corresponding page. The ToC is represented by folder and stream objects. The folder objects offer dictionary-like access to their children, while stream objects store the IDs of the pages belonging to them. The library uses NumPy (Harris et al., 2020) to improve the reading speed and memory efficiency of the arrays storing page IDs, amplitudes, and timestamps. To keep the library portable, NumPy is the only required dependency. The functionality to convert a recording to the Neo structure is implemented as an optional dependency. As different experiment set-ups may produce different structures in the DAPSYS file, there is no “universal” converter. Instead, the library provides an abstract base class for Neo converters, which offers functions for common conversions (i.e., text stream to event). Based on this class, additional converters may be implemented for different ToC structures.

#### 3.2.1. Verification

As described in section 2.3.1, we compared the CSV data exported by DAPSYS with the data read by PyDapsys. Depending on the type of stream being exported to CSV, the resulting file contains different values:

- Waveform streams: Contain both the timestamps for each data point and its signal value. Both timestamp and signal values have a precision of 6 decimals.
- Text streams: Contain the timestamps for each text with a precision of 4 decimals and the text itself.

Across all files used for testing, 284,453,786 individual floating-point values were compared, of which 3,009,074 values differed. The maximum difference was 0.00001. As this is exactly the precision offered by waveform CSV-exports, it is most likely a result from rounding errors and not a systemic error in the PyDapsys implementation. There were no differences in the text data.

#### 3.2.2. Performance testing

As seen in Table 1, storing data in NIX/H5 with Neo had no significant impact on file sizes compared to the original file, whereas the CSV increased the file size by factor 4. PyDapsys reliably outperformed DAPSYS in the time required for exporting a file by more than factor 97 (see Table 2).

## 4. Discussion

In order to make electrophysiological recordings obtained with the DAPSYS DAS available to other systems in our lab, we implemented the open-source Python library “PyDapsys.” The library has functionality for reading data from DAPSYS files and offers built-in functions to automatically load read data into the structure defined by the Neo library, from where it can be exported to NIX and other data formats, which are used by the neuroscience community and can be read by various other software solutions. By offering direct access to the data stored in DAPSYS files, rounding errors that may occur when exporting the data to CSV are avoided, thus improving the accuracy and quality of subsequent analyses. The library outperforms the DAPSYS CSV export, both in export duration and size of the exported files, while additionally not being dependent on DAPSYS itself. Currently, the usage of the PyDapsys library requires a certain level of programming experience. To make the library available for a more general audience, we are working on implementing a GUI (graphical user interface).

While DAPSYS is not used very commonly, it should be seen as a representative of many domain-specific proprietary formats, which are used in neuroscientific research. FAIR data handling principles require the accessibility and interoperability of data, and the opening of proprietary formats is a necessary step to ensure those qualities (Berens and Ayhan, 2019). We expect the presented process of analyzing the files with the “ImHex” software and modifying the parameters to understand their internal structure to be useful for other research groups, who are facing similar challenges. It is important to note that the DAPSYS file format does not utilize any compression or encryption. Reverse engineering compressed or encrypted data would have made the process significantly more difficult.

In general, our case highlights the importance of proper procedures to ensure long-term access to experimental data. In the microneurography community, the experiments are complex, and many data sets are unique due to rare genetic mutations of the patients. Moreover, guaranteeing reliable access and unification of data also simplifies collaboration between research groups. Therefore, ensuring FAIR principles allows us to optimize the research benefit derived from the data.

The appropriate processes should ideally be put in place early on to ensure that data is available in open formats. For example, if the formats cannot be read using open software, this could include manual exporting new data to open formats once a week to avoid forming a backlog and potentially losing access to large quantities of non-exported data if the original software is not available anymore.

Open science and FAIR principles are becoming more and more widely accepted in academia and in neuroscience in particular. However, at the current stage of ongoing works, it is important to include smaller communities in the discussion, as the popularity of the specific software and hardware solution influences the motivation of the vendors to provide open off-the-shelf solutions. PyDapsys alongside more general emerging approaches, such as MARBLE, serves as an example of a possible solution for these research communities.

## Data availability statement

The data analyzed in this study is subject to the following licenses/restrictions: the hospital regulations limit open data sharing. Data is available upon reasonable request. Requests to access these datasets should be directed to BN, bnamer@ukaachen.de.

## Ethics statement

The studies involving humans were approved by the Ethics Boards of the University Hospital RWTH Aachen and Friedrich-Alexander-University of Erlangen-Nürnberg. The studies were conducted in accordance with the local legislation and institutional requirements. The participants provided their written informed consent to participate in this study.

## Author contributions

PK developed the software and drafted the manuscript. AT supervised the work on microneurography data. APG and APM supervised the software-development work. RR, BN, and EK supervised the project. All authors substantially revised the manuscript.

## Funding

This work was partially funded by the Excellence Initiative of the German Federal and State Governments G:(DE-82) EXS-SF-SFDdM013 and also supported by the IZKF TN1-6/IA 532006. BN was supported by a grant from the Interdisciplinary Center for Clinical Research within the Faculty of Medicine at the RWTH Aachen University and the German Research Council DFG NA 970 3-1, DFG FOR 2690 project 6.

## Conflict of interest

APM was employed by Forschungszentrum Jülich GmbH.

The remaining authors declare that the research was conducted in the absence of any commercial or financial relationships that could be construed as a potential conflict of interest.

## Publisher’s note

All claims expressed in this article are solely those of the authors and do not necessarily represent those of their affiliated organizations, or those of the publisher, the editors and the reviewers. Any product that may be evaluated in this article, or claim that may be made by its manufacturer, is not guaranteed or endorsed by the publisher.

## Footnotes

^1^DICOM: Digital Imaging and Communications in Medicine, Medical Imaging Technology Association (MITA), https://www.dicomstandard.org.

^2^Data Acquisition Processor System (DAPSYS), Brian Turnquist, http://dapsys.net.

^3^MARBLE software project, Steffen Brinckmann et al., https://gitlab-public.fz-juelich.de/marble.

^4^ImHex, Nikolaij “WerWolv” Sägesser, https://github.com/WerWolv/ImHex.
